# Collargolum and Unguentum Crede in Gonorrhea and Its Sequelae

**Published:** 1904-04

**Authors:** 


### Collargolum and Unguentum Crede in Gonorrhea and its Sequelae.

Kornfeld, Vienna, in his recently published brochure, Gonorrhea and Matrimony, (Franz Deuticke, Vienna and Leipsic, 1904,) recommends collargolum, unguentum Credé and itrol for the chronic follicular inflammations of the glandular structures around the urethral orifice. They have done him good service in the indurations of the frenum that these affections so frequently leave behind; and in periurethral nodosities and bands, when not too old.

The author is a decided opponent of the use of ice in the acute stage of unilateral or bilateral epididymitis. It quiets the patient momentarily, but its effects upon the resultant induration of the gland are bad, and even a purely expectant treatment is preferable. When the acute symptoms have subsided especially, unguentum Credé has been found very valuable. It promptly and efficiently promotes the resorption of the inflammatory exudation. Satisfactory results are seen from collargolum instillations and injections (¼ per cent. to 1 per cent.) in very recalcitrant gonorrheas, the cause of whose persistency is the involvement of the prostate in the creeping inflammatory process.

Unguentum Credé, thinly spread on linen, is applied two or three times daily and covered with a moist dressing, which aids its absorption. This is of the greatest value for preventing induration, distortion or obliteration of the vas deferens and for preserving virility. The silver salve plays a prominent role in the therapy of other gonorrheal complications and residual processes, as in acute and chronic prostatitis, in the form of suppositories containing ¾ to 2¼ grains of collargolum or 4 grains of unguentum Credé.

Kornfeld lays special stress upon the use of these remedies in female gonorrhea. Especially unguentum Credé has proved to be an admirable absorbefacient and disinfectant for puerperal exudates, and in puerperal sepsis. When employed either externally as an inunction or by enema, the results from its use have been nothing less than brilliant. In the gonorrheal affections of women uterine lavage and vaginal and cervical tamponades with these remedies have a most excellent germicide and desiccant effect, as Kornfeld's own abundant experience proves. He deems it his duty to call attention to the eminent efficacy of the *globule vaginales* made of unguentum Credé in promoting the energetic absorption of painful pelvic exudates.

As regards systemic infection with the gonorrheal virus, gonococcopyemia, the author obtained surprisingly good results from collargolum enemata in three personal cases, which in spite of desperate symptoms resulted in complete recovery. The author emphasises the importance of the silver salts and of collargolum in these affections, and calls them sovereign therapeutic agents. Even in true uterine gonorrhea permanent collargolum irrigation for two to four days is of the greatest value.

